# Trajectory-based characteristic analysis and decision modeling of the lane-changing process in intertunnel weaving sections

**DOI:** 10.1371/journal.pone.0266489

**Published:** 2022-04-04

**Authors:** Yi Zhao, Zhiqi Wang, Yuxuan Wu, Jianxiao Ma

**Affiliations:** College of Automotive and Traffic Engineering, Nanjing Forestry University, Nanjing, Jiangsu, China; Tongji University, CHINA

## Abstract

Existing lane-changing models generally neglect the detailed modeling of lane-changing actions and model lane-changing only as an instantaneous event. In this study, an intertunnel weaving section was taken as the background, the lane-changing duration and distance in the lane-changing process were taken as the main research objects. The detailed modeling of a lane-changing action was emphasized. Aerial videos of intertunnel weaving sections were collected, and accurate vehicle trajectory data were extracted. Basic data analysis shows that the lane-changing duration has a lognormal distribution and the lane-changing distance has a normal distribution. To analyze the difference of the lane-changing behavior characteristics in different lane-changing environments, based on the lead spacing and lag spacing in the target lane, a hierarchical clustering algorithm was applied to classify the lane-changing environment into six different types. Then, a deep neural network regression model was applied to model the lane-changing process for each environment type. The results show that the horizontal distribution, vertical distribution and statistical characteristics of the lane changing points under different lane-changing environments are significantly different. The prediction accuracy of the lane-changing distance after classification is improved by at least 61%, and the prediction accuracy of the lane-changing duration after classification is improved by at least 57%. It is also found that lane-changing behavior characteristics with large or small lag spacing are easier to predict, while in the other cases, the randomness of the lane-changing behavior characteristics is more obvious. The research results can be incorporated into lane-changing decision assistance systems and micro traffic simulation models to make the assistance system safer and more effective, and the simulation outputs should be more realistic and accurate.

## 1. Introduction

**Fig 1:**
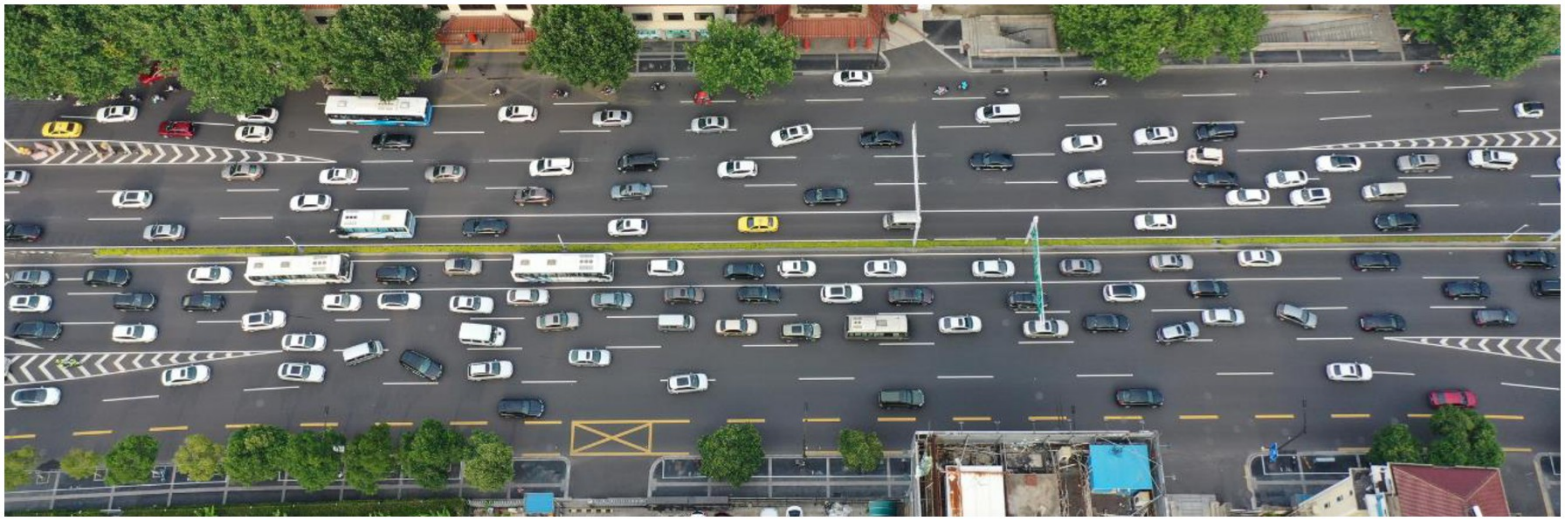
Field picture of the studied area.

The number of tunnels has increased dramatically over the past three decades, especially in metropolises [1], and the tunnels in a city are mainly road tunnels. The enclosed environment of a tunnel can provide a smooth and undisturbed traffic flow environment, but the traffic exchange between a tunnel and a surface road can form a fixed interweaving zone, as shown in Fig 1, which will be referred to as “intertunnel weaving sections” [2] in the following paper. In-tunnel and off-tunnel vehicles need to interact in a compact space. As a result, heavy weaving behaviors tend to develop into potential traffic hazards and traffic bottlenecks. Intertunnel weaving sections have much in common with other freeway or expressway weaving regions, but they also have their own unique geometric designs, driver behaviors, etc.

Weaving sections have always been the focus of traffic safety. And lane-changing behavior is one of the most important factors that affects traffic safety in weaving sections. According to statistics, 80% of traffic accidents in urban tunnels are caused by lane-change behavior [3], and lane-changing accidents in intertunnel weaving sections account for 32.6% [2]. Many rear-end accidents are also caused by lane-change behavior. Besides, the research on lane-changing behavior has a wide range of application requirements, such as improving traffic demand management [4], emergency management and infrastructure design [5] base on lane-changing behavior characteristics and capacity estimation. Traffic behavior modeling is also the basis of constructing traffic simulation environment [6] and provides research tools to the research of assisted driving [7] and automatic driving [8]. To this end, this paper focuses on analyzing the behavioral characteristics and decision-making process of intertunnel weaving sections. Therefore, a reference for the calibration of lane-changing behavior parameters of a safe driving assistance system, microtraffic behavior and safety simulation models, emergency management and infrastructure design are provided. Existing studies on lane-changing behaviors can be generally divided into three categories: traffic behavior modeling and behavior characteristics analysis.

Traditional lane-changing behavior models include the LWR lane-changing model, cellular automata model, probability selection model, and regression analysis model. For example, Pan [9] proposed a meso-multilane model to simulate mandatory and arbitrary lane-changing behavior by studying the minimum gap acceptance criterion and allocation of spaces for merging flows. Hidas [10] performed modeling and simulation studies on vehicle interaction behaviors in a weaving section and analyzed speeds, gap acceptance and conflict resolution in the lane-changing process. In addition, existing lane-changing models mainly focus on single lane-changing behavior. Zhe Meng [11] proposed that continuous lane-changing behavior exists in ramp areas, but there are few studies on continuous lane-changing behavior. In recent years, artificial intelligence algorithms have gradually been used to model driver behaviors. For example, Wang Junyan [12] used an RBF neural network to establish a vehicle lane-changing timing decision model, which could predict the safety of an upcoming lane-changing action to ensure the safety of drivers and vehicles. Liu Yalong [13] used the BP neural network, the support vector machine and the random forest model to establish a data-driven decision model for free lane changes. Zhang [14] used a deep learning method to model lane-changing and car-following behaviors. Compared with other road sections, driver interaction behavior in weaving sections are more complex, and artificial intelligence algorithms have become an important method for traffic flow analysis in these areas. However, existing lane-changing models emphasize the decision-making aspects of the task, generally neglect the detailed modeling of the lane-changing action itself and model it only as an instantaneous event.

There are two key parameters during the lane-changing implementation period: the duration and distance of lane-changing behavior. However, only a limited number of studies that address the duration and distance parameters of lane-changing have been presented in the literature. A study of lane-changing duration and distance was first performed in 1970. Different means, including aerial photographs, eye markers, helmets, observers, and simulators, have used to obtain lane-changing behavior data. In most cases, human observers or obtrusive equipment may have affected drivers’ behavior and biased the results. Data collected by using a driving simulator [15] are not representative of naturalistic driving, which may negatively affect the realism of the driving experience and the fidelity of the data collected. It is therefore not surprising that some researchers emphasize the lack of on-road lane-changing data as an important limitation to studies that use these data to develop driver assistance systems and evaluate weaving sections. With the development of technology, more accurate lane-changing data are currently available. A large set of trajectory data [16] that was collected by high-mounted video cameras in naturalistic driving conditions was used by Toledo [17] to study lane-changing behavior. It was found that the passenger car lane-change duration has a lognormal distribution. Wang Xuesong [18] analyzed the cut-in behavior of following vehicles based on realistic driving behavior data and found that the cut-in duration was approximately 5.7 seconds and had a lognormal distribution. In terms of study scenarios, freeways are often taken as the background for the study of weaving behavior, while urban expressways or other urban roads are rarely used. Fu Xinsha [19] found that the lane-changing duration on freeways followed a lognormal distribution and was significantly related to traffic conditions. Yu Qiang [20] found that the average lane-changing duration on freeways is 6.09 seconds, and the average lane-changing distance is 148.08 meters, which conform to a lognormal distribution. There are also some special scenarios of lane-changing behavior, such as behavior in construction areas. Wen Jiaxian [21] modeled vehicle merging behavior in work zone merging areas during a merging implementation period from the safety perspective. It is worth noting that because of the difference in the definitions of the initiation and completion of lane changes, the analysis results differ in the various studies on the average lane-changing duration and distance.

In summary, the above discussion clearly indicates that the literature regarding vehicle lane-changing behavior in intertunnel weaving sections is rather limited. Therefore, the objective of this study is to analyze lane-changing behavior characteristics and model vehicle lane-changing behavior in intertunnel weaving sections during the merging implementation period while not treating lane-changing behavior as an instantaneous event. Trajectory-based lane-changing duration data and real-time vehicle status data were collected. Theoretically, a larger gap between the front and rear vehicles in the target lane may produce more relaxed and free lane-changing behavior, which may result in longer lane-changing duration times and distances. Considering that different lane-changing behaviors may be implemented depending on the various gap sizes, the type of lane-changing behavior should be analyzed. Hence, it is more realistic to model vehicle lane-changing behavior according to different conditions. If the lane-changing decision rules during the lane-changing implementation period are incorporated in a micro traffic simulation model, a more realistic and accurate traffic flow environment of intertunnel weaving sections can be reproduced. Hence, lane-changing decision assistance systems can be more effective, safe, and helpful.

## 2. Data collection and statistical analysis

### 2.1. Geometric design and data extraction

**Fig 2:**
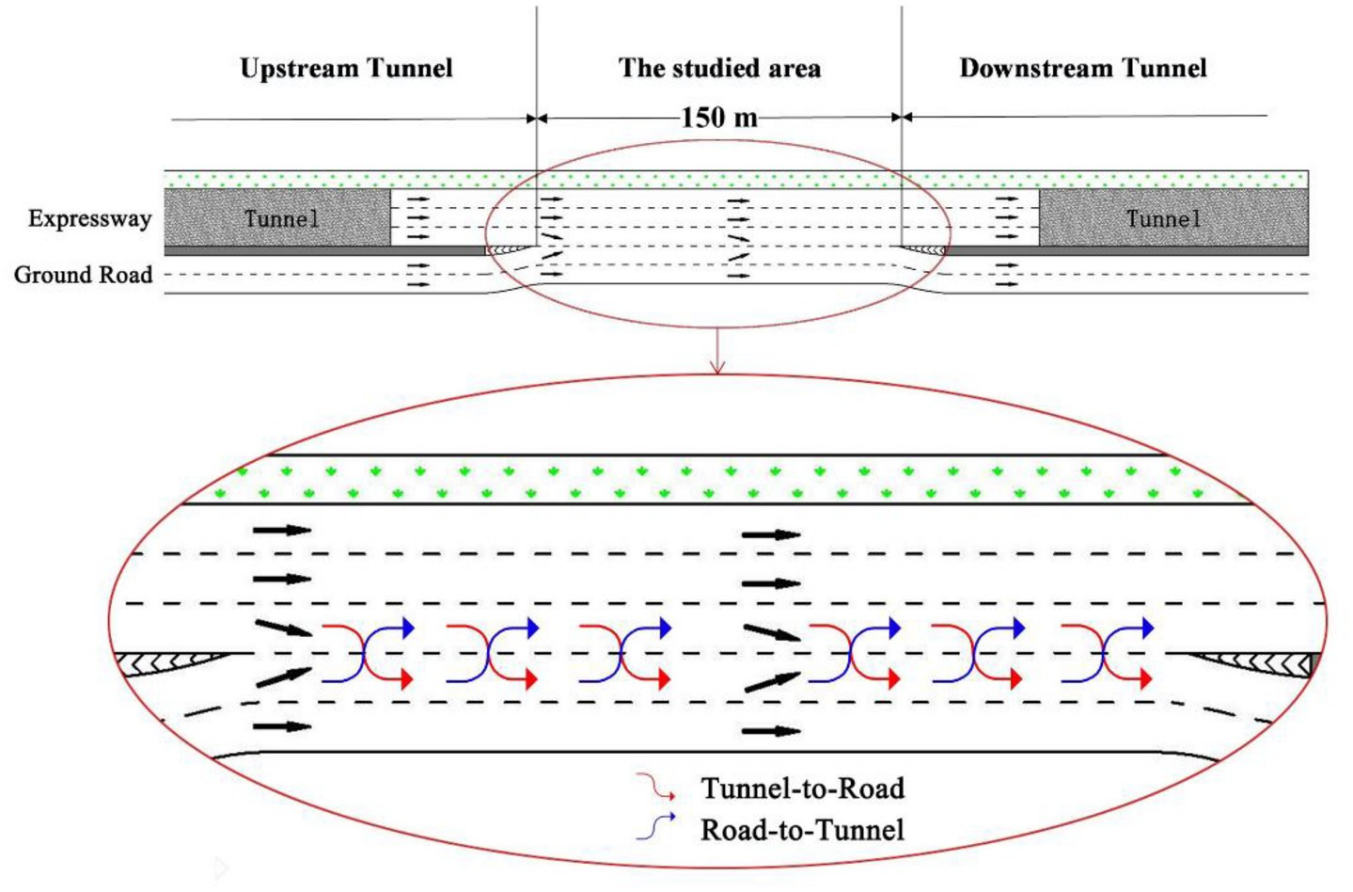
Geometric structure and two weaving traffic streams of weaving sections.

Field data was collected in Nanjing, China. The geometric structure of the weaving section is illustrated in Fig 2. This site is an ordinary road section where no permits are required for the access. For simplification, the mainline connecting upstream and downstream tunnels are called the tunnel expressway, while the surface road connecting to tunnels are referred to as the ground road. There are three lanes for the tunnel expressway and two lanes for the ground road, and the width of one lane is 4m. The tunnel expressway is straight, and the length of the weaving section is 150m. A single dashed line separates the tunnel expressway and the ground road. The single dashed line indicates that weaving vehicles can change lanes at any point. Two weaving traffic streams, i.e., the road-to-tunnel flow and tunnel-to-road flow, are denoted as the merging flow and diverging flow, respectively.

**Fig 3:**
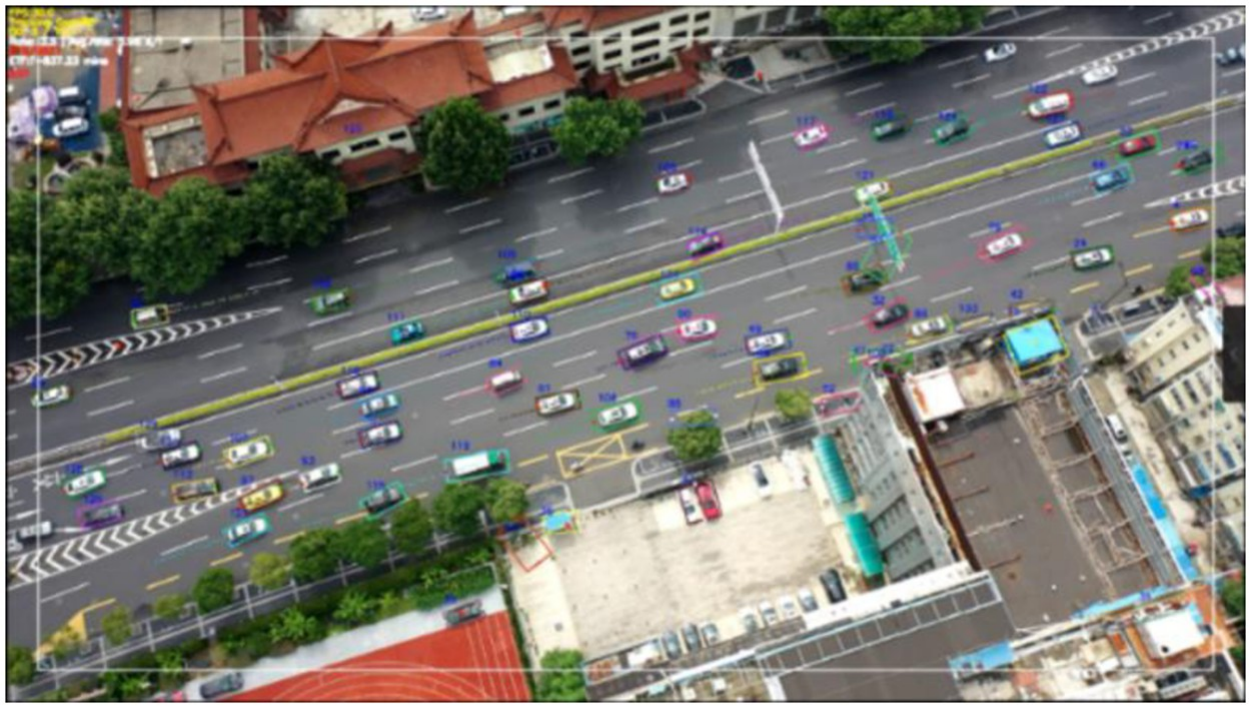
Video processing by using the automated roadway conflicts identification system.

An unmanned aerial vehicle (UAV), Phantom 4 Pro V2.0, was used to take videos of the study area. The UAV hovered at an altitude of 200 meters above the study area to obtain a clear view. All videos were taken under good weather conditions. The videos were taken during weekdays in Jan 2021, a total of two hours of videos were used in the study. Then, the vehicle trajectory data was extracted by using the Automated Roadway Conflicts Identification System [22], which was provided by the UCF Smart & Safe Transportation Lab. A screenshot of the video processing is shown in Fig 3. Video stabilization technology is incorporated in the system to keep the perspective stable. The system can display 30 frames per second and extract the pixel coordinates of every vehicle’s central point in the video. Then, an affine transformation is applied to convert the pixel coordinates to Cartesian coordinates, which take the straight direction of the vehicle as the X-axis and the direction perpendicular to the lane as the Y-axis. The raw data information includes the vehicle ID, frame number, Cartesian coordinates of the vehicle location, vehicle length, and vehicle model.

### 2.2. Descriptive analysis of the interweaving process

**Fig 4:**
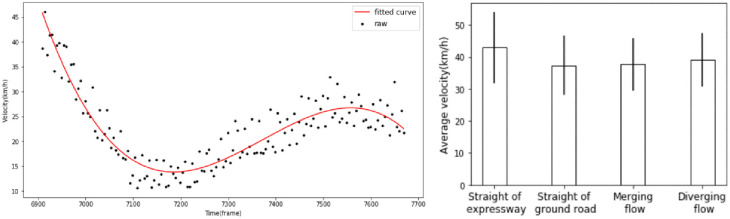
Velocity fitting diagram and velocity statistical feature diagram.

Data from 6,000 vehicles, which contain 1,400 weaving vehicles, were recorded. Of the lane-changing vehicles, 42 (3.0%) are classified as heavy vehicles, and the rest are passenger cars. There is no vehicle speed information in the raw data, and the space-mean speed is taken as the average speed of vehicles in each subsection in this study. However, the smaller the subsection length is, the greater the velocity error. By increasing the subsection length, it is found that the speed value will reach the steady state when taking 5 frames as the subsection length, as shown in Fig 4. By curve fitting the speed of every 5 frames, the variation rule for the vehicle speed can be determined and the vehicle speed value for each frame can be assigned. The average speed of different passing vehicles types in the study area is shown in Fig 4.

The average speed of straight-flow traffic on expressways was 42.9 km/h (the deviation was 11.1), and the straight-flow traffic on ground roads was 37.2 km/h (the deviation was 9.2). The average speed of merging-flow traffic was 37.6 km/h (deviation was 8.2), and that of diverging-flow traffic was 39.1 km/h (the deviation was 8.3). The average speed of traffic on intertunnel weaving sections is obviously lower than that on freeways. There is a certain speed difference between the expressway and the ground road, while the weaving-flow traffic speed is similar to the direct traffic speed on the ground road.

**Fig 5:**
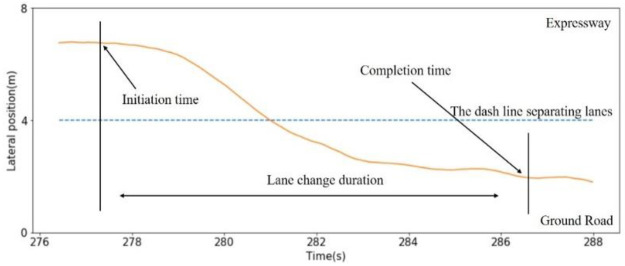
The initiation and completion points of lane-changing behavior.

**Fig 6:**
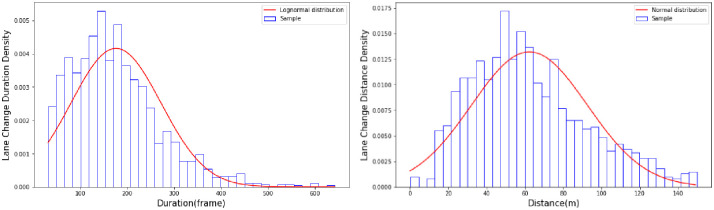
Statistical histogram of the lane-changing duration and distance.

Considering the vehicle trajectory is not stable, for each lane-changing behavior, the initiation and completion points of lane-changing behavior are identified by combining the lateral movement and the position in lane of the subject vehicle. The initiation point is defined as the time and space instances when the lateral movement has started and the center of the vehicle is one third of one lane width away from the target lane. The completion point is defined as the instance when the lateral movement is not over and the center of the vehicle is one third of one lane width away from the initial lane. The one third of one lane width is selected to ensure the subject vehicle is within the current lane and will not affect the traffic in the adjacent lane. Fig 5 demonstrates these points with the trajectory of one of the vehicles in the dataset. The lane-changing duration is the time duration between the lane-change initiation and completion, and the lane-changing distance is the movement length in the lane direction from the initiation and completion points of the lane change. Statistical histograms of the lane-changing duration and distance are shown in Fig 6. The lane-changing duration has a lognormal distribution that is fitted to the data (μ = 176.158 and σ^2^ = 95.968), and the lane-changing distance has a normal distribution (μ = 62.364 and σ^2^ = 30.217). The lognormal and normal distributions guarantee that lane-change durations and distances are nonnegative.

**Fig 7:**
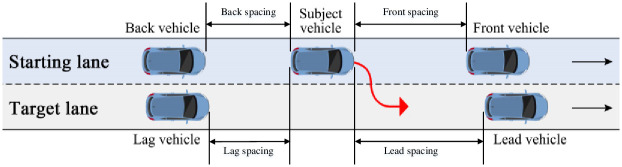
Definition of the parameters between the subject vehicle and other vehicles.

**Fig 8:**
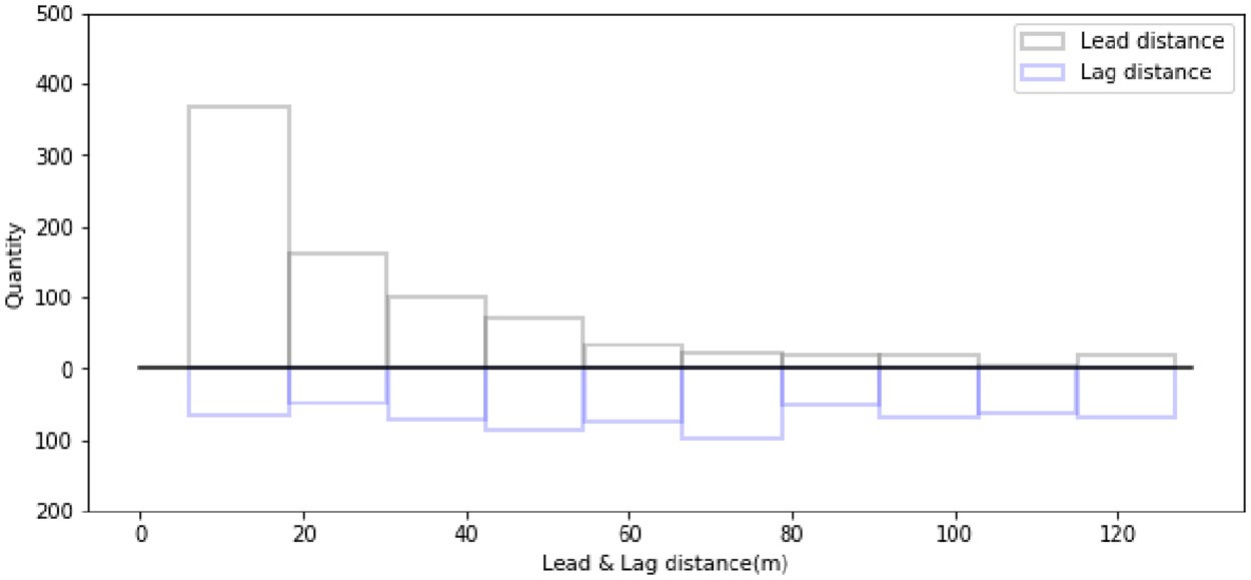
Statistical histogram of the lead and lag distance.

According to driving experience, lane-changing decisions and behavior characteristics are directly affected by the available gap in the target lane. When the available gap is large, the lane-changing distance maybe long. When the gap is small, especially when the distance to the lead vehicle in the target lane is relatively close, the lane-changing distance is restricted. For each lane-changing vehicle, the spacing between the subject vehicle and the car in front of them in the target lane at the start of lane-changing process is defined as the lead spacing, and the spacing between the subject vehicle and the car behind them in the target lane is defined as the lag spacing. Both the lead and lag spacing refer to the distance between the front of a vehicle and the back of another vehicle, not including the vehicle length. The subject vehicle, the vehicles around it, and the variables that define the relations between them are shown in Fig 7. The statistics for the lead and lag spacing are shown in Fig 8. In addition, lead and lag distances that are greater than 120 meters are not shown in the figure. Most of this distance occurs because there is no lead or lag vehicle.

## 3. Feature analysis of lane-changing process

### 3.1. Lane-changing environment classification

The lane-changing process is influenced by many factors, such as the geometric design, vehicle and driver characteristics, and surrounding traffic environment. The geometric design includes the vertical and horizontal road lines and pavement quality. The vehicle and driver characteristics include the performance of the vehicle, drivers’ driving habits and drivers’ characteristics. The surrounding traffic environment includes the driving conditions of the vehicles before and after the target vehicle changes lanes. For intertunnel weaving sections, the geometric design is a fixed environment, which is described in detail in the previous section. Weaving vehicles are mainly passenger cars, and there is no objective classification standard for driver characteristics. Neither of them can be used as the dividing standard for the traffic operation environment. Therefore, this paper intends to classify traffic environment types based on the characteristics of the traffic environment. The difference in lane-changing behaviors in different environments should be analyzed to accurately describe the lane changing process. Based on driving experience, this paper assumes that the lead and lag distance have a significant impact on the lane changing process of the target vehicle. The hierarchical clustering algorithm was applied to classify the weaving environment of lane-changing behavior.

**Fig 9:**
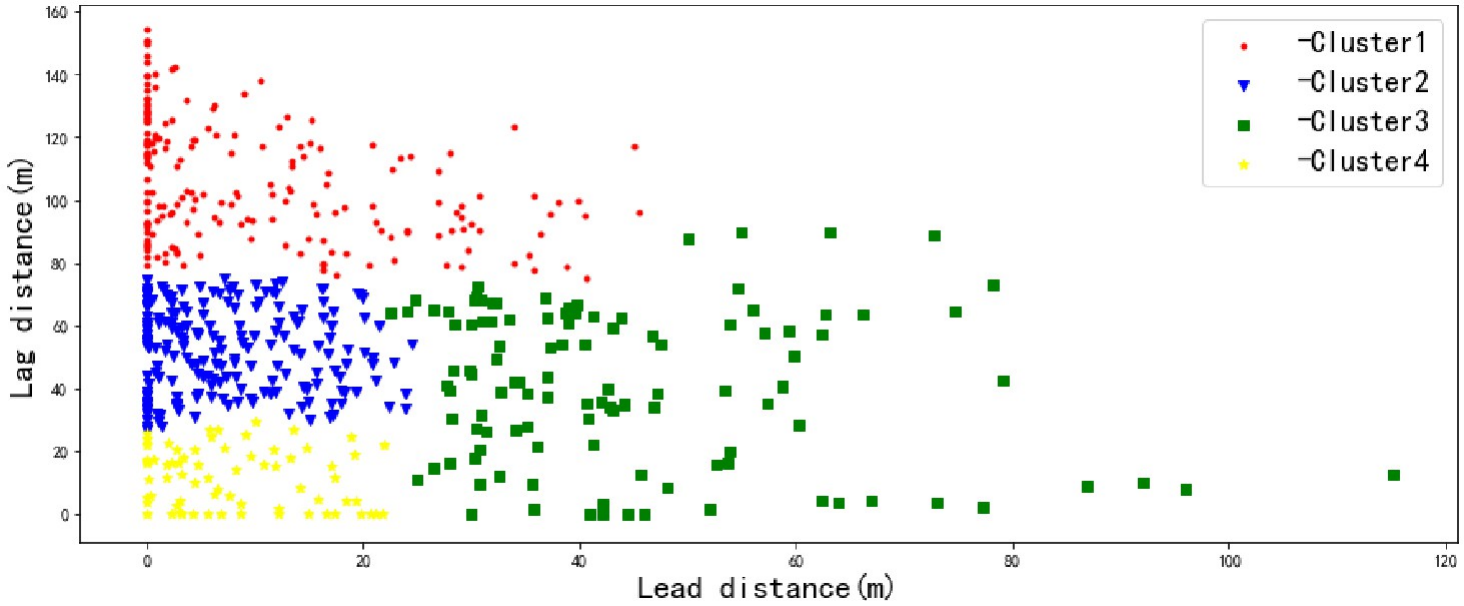
Hierarchical clustering analysis results.

Considering that the conditions in which there is no lead vehicle (total number is 255) or lag vehicle (total number is 326) are special environments, cluster analysis excluded these two lane-changing environments. Clustering analysis of the lead and lag vehicles is realized by Python, and the results are shown in Fig 9.

As seen from the figure, the lane-changing environments should be divided into 4 categories according to the lead and lag distances. Considering that the environment types with no lead vehicle or lag vehicle were not included in the cluster analysis, the weaving environment of lane-changing behavior can be divided into the following six categories. The sample sizes of the six types of weaving environments are shown in Table 1.

- The first type is Cluster 1 in Fig 9; the lead distance was discrete, and the lag distance was large.
- The second type is Cluster 2 in Fig 9; the lead distance was small, and the lag distance was moderate.
- The third type is Cluster 3 in Fig 9; the lead distance was large, and the lag distance was moderate.
- The fourth type is Cluster 4 in Fig 9, and both the lead distance and the lag distance were small.
- The fifth type had no lead vehicle, and the lag distance was small.
- The sixth type had no lag vehicle, and the lead distance was discrete.

**Table 1:** Sample sizes of different types of weaving environments.

Type of weaving environment	The first type	The second type	The third type	The forth type	The fifth type	The sixth type
Sample size	255	279	171	114	255	326

### 3.2. Lane-changing duration and distance feature

**Fig 10:**
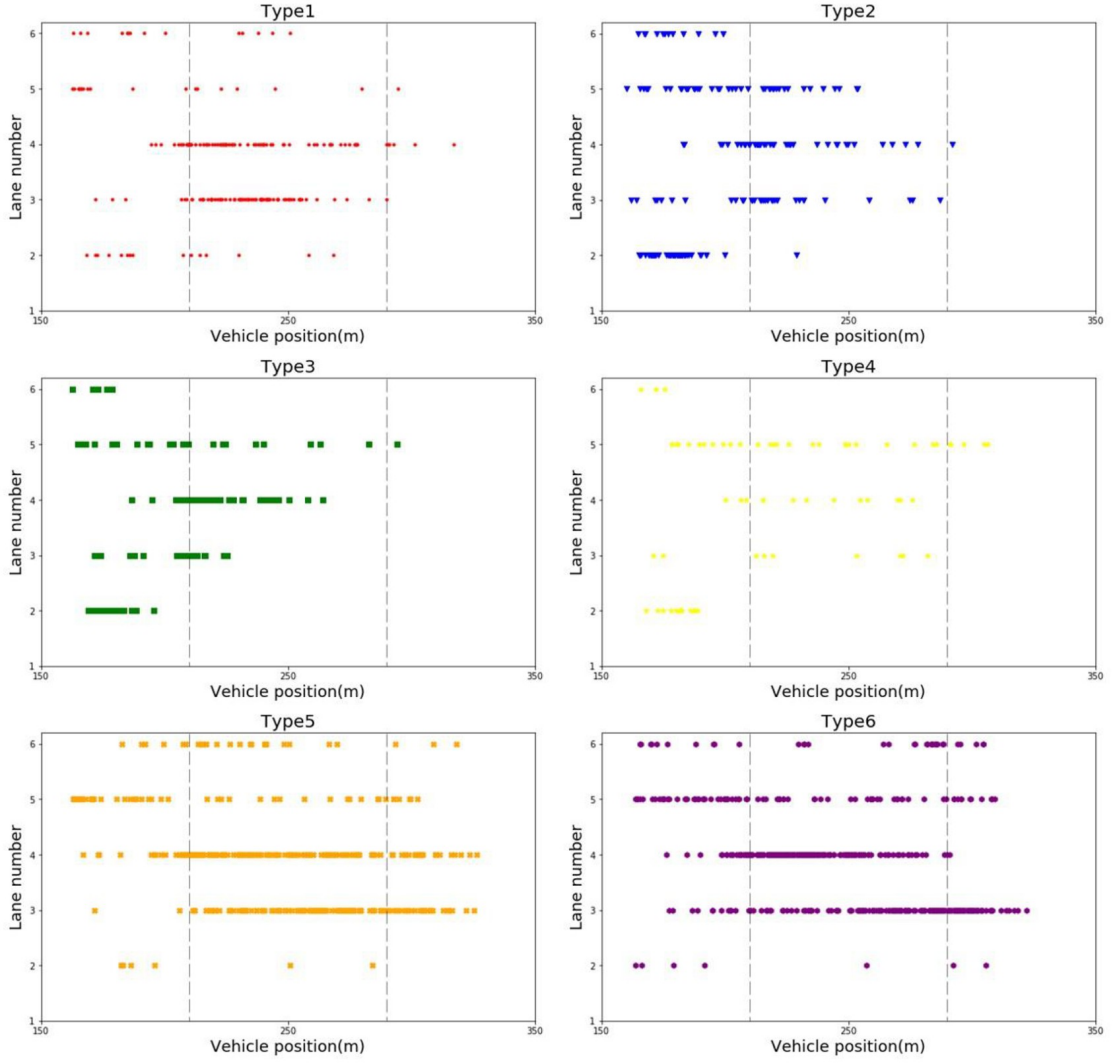
The distribution of the six environment types in the weaving section.

The distribution of the six types of weaving environments on the lanes in the weaving section is shown in Fig 10. The ordinate represents the lane number, where 4 to 6 represent 3 expressway lanes, 1 to 3 represent 3 ground road lanes, and 1 stands for the bus only lane. The abscissa indicates the locations of the lane-changing starting points for different types of weaving environments in the weaving section. The following distribution characteristics can be concluded from the figure.

- Since the lane-changing is prohibited in the tunnel, vehicles in the middle lane of the expressway (Lane 5) must converge to the outermost lane (Lane 4) as soon as possible after exiting the tunnel if they want to exit the expressway. Therefore, the left rear of the lane-changing point of Lane 4 is dense.
- For types 1 to 4, lane-changing points are mainly located in the first half of the weaving section. The merging and diverging behaviors tend to be completed as soon as possible, which decreases the lane-changing occurrences in the second half of the weaving section.
- For type 5 and type 6, the lane-changing point distributions in the first half and the second half of the weaving section have no significant differences. The main reason for this phenomenon is that the two lane-changing environments are very conducive to the completion of lane-changing behavior. Therefore, when a lane-changing opportunity arises, vehicles use that opportunity to change lanes.
- There were significant differences in the types of lane-changing behaviors, the distributions in the different lanes, and the distributions in the same lane for types 1 to 4.

**Fig 11:**
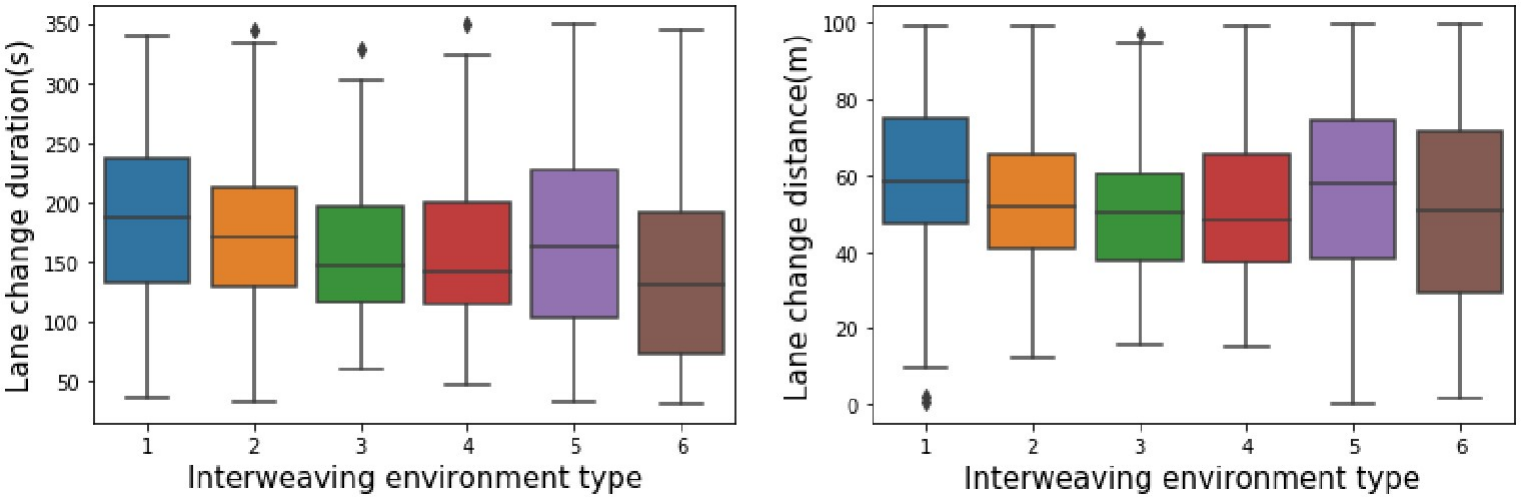
Box diagrams of the lane-changing duration and distance.

For the six different weaving environment types, the lane-changing duration and distance behavior data in the different environments were extracted, and a corresponding box diagram was drawn, as shown in Fig 11. The following features can be seen in the box diagram.

- There are significant differences in the median, upper quartile and lower quartile of the lane-changing behavior characteristics in the six types of weaving environments. This indicates that there are significant differences in the lane-changing behavior characteristics of vehicles in different weaving environments.
- The boxes of types 5 and 6 are larger than those of the other 4 types, indicating that the lane-changing durations and distances of these two types are relatively discrete, while these two features of the other 4 types are relatively concentrated.
- The medians of type 3 and type 4 are close to the quartile values, indicating that the lane-changing behavior has a skewed distribution in this environment. The median lane-changing behavior in each of the other environments is almost in the middle of the diagram, so it can be considered that these lane-changing behavior characteristics meet a normal distribution.
- There are some outliers in the lane-changing behavior data in the first four types of environments, and the outliers are removed in subsequent modeling.

## 4. Decision modeling of the lane-changing process

### 4.1. Deep neural network regression modeling

**Fig 12:**
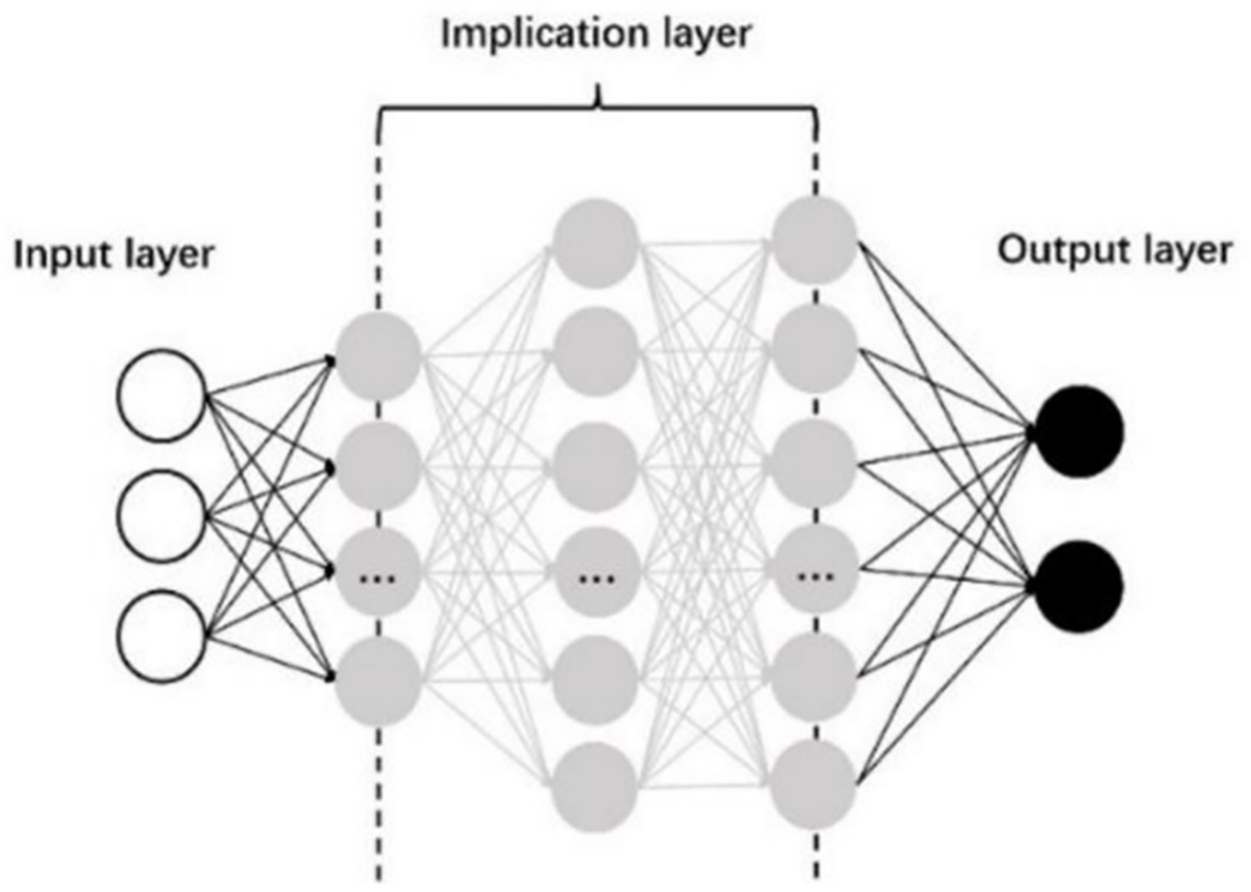
Structure of the weaving model based on a neural network.

A neural network is a nonlinear complex network system composed of a large number of processing units that are similar to biological neurons and can simulate the intelligent behavior of biological neural networks through a certain mathematical model; they can be used to solve some intelligent information processing problems that traditional algorithms cannot solve. Compared with traditional neural networks, deep neural networks (DNNs) have deeper layers and stronger feature expression abilities. The structure of a DNN consists of three layers, as shown in Fig 12. The first layer is the input layer, which stores the input variables, and each node represents a variable. The second layer is the hidden layer, which is the core part of the deep neural network and mainly includes a number of hidden layers, the number of neurons corresponding to the hidden layer and the activation function. The structure of the hidden layer can be determined by adjusting these variables to better complete deep learning. The third layer is the output layer, which outputs the variable values.

The learning process of a deep neural network is composed of signal forward propagation and error back propagation. For forward propagation, the input sample *X*_*i*_ = (*x*_1_, *x*_2_, ⋯, *x*_*n*_) from the input layer passes to the output layer after being processed by the hidden layer, and the output vector is *Y*_*i*_ = (*z*_1_, *z*_2_, ⋯, *z*_*n*_). If the weight vector between the input layer and hidden layer is *V*_*j*_ = (*V*_1_, *V*_2_, ⋯, *V*_*p*_) and the weight vector between the hidden layer and output layer is *W*_*k*_ = (*w*_1_, *w*_2_, ⋯, *w*_*p*_), then the output of the hidden layer can be expressed as follows.


$$z_{j}=f_{1}(\sum_{k=1}^{n}V_{jk}x_{k}+\theta_{j}),j=1,2,\cdots ,p$$ (1)

The output of the output layer can be expressed as follows.

$$O_{i}=f_{2}(\sum_{j=1}^{p}z_{j}w_{ij}+\theta_{i})$$ (2)

where *f*_1_ and *f*_2_ are activation functions and *θ*_*j*_ and *θ*_*i*_ are the intercepts of the two functions. *V*_*j*_ represents the weight vector of neuron j in the hidden layer, and *w*_*k*_ represents the weight of neuron k in the output layer. If the output value *O*_*i*_ of the output layer meets the expected output requirement *y*_*i*_, the calculation ends. Otherwise, the process enters the error backpropagation stage.

When the error is transmitted back, the error between the network output and the expected output $e_{i}=\frac{1}{2}{\left(O_{i}-y_{i}\right)}^{2}$ is transmitted back through the hidden layer. The error is apportioned to neurons of each layer, and the error signal of the neurons in each layer is obtained. The error signal is the basis for correcting the weight of each neuron. In the process of correction, the purpose is to reduce the error, and the weight updating formula with learning efficiency α is obtained by adjusting the direction of the negative gradient of the neuron weight. The weight updating formula is as follows.


$$w_{ij}(t+1)=w_{ij}(t)-\alpha (O_{i}-y_{i})f_{1}$$ (3)

$$V_{jk}(t+1)=V_{jk}(t)-\alpha (O_{i}-y_{i})w_{ij}f_{1}^{\prime }x_{k}$$ (4)

The learning process of a DNN is a weight adjustment process that is repeated for each layer and combined with the signal forward propagation and error back propagation processes. This process continues until the error of network output is reduced to an acceptable degree or until the process reaches the preset learning time.

### 4.2. Decision model training and testing

In this paper, the model structure shown in Fig 12 is used to establish the DNN-based weaving process decision model. Considering that the lane-changing duration and distance in the weaving process are affected by vehicle’s state and the surrounding environment, the current speed V of the target vehicle, lead distance and lag distance are taken as the input variables of the input model. At present, there is no clear way to select the most appropriate neural network structure. After training and testing by adjusting the parameters of the hidden layer, the hidden layer number is set as 3. According to experience, the optimization effect is better when the number of neurons is set as 2^*n*^. The numbers of neurons in the hidden layers are set as 64, 64, and 128 based on testing and comparison. The output variables are the lane-changing duration and distance. The neural network model requires continuous training by constantly updating the weight matrix in the training process. In this study, the commonly used ReLU function is selected as the activation function to avoid the weight increment approaching 0 due to small derivative multiplication and to effectively solve the problem of gradient disappearance. The Adam algorithm is used to optimize the algorithm, and the learning efficiency is specified as 0.0001. The mean square error (MSE), that is, the average sum of squares of the difference between the predicted value and true value, is selected as the performance index of neural network training. The smaller the MSE value is, the better the data prediction effect is.


$$MSE=\frac{1}{m}\sum_{i=1}^{m}{\left(y^{\left(i\right)}-{\overset{-}{y}}^{\left(i\right)}\right)}^{2}$$ (5)

For the weaving behavior data before classification and the six types of weaving behavior data after classification, the DNN model was used for training and testing. Here, 70% of the data of each type are used for model training and 30% are used for model testing. The weaving behavior data of each type are shown in Table 2.

**Table 2:** Weaving behavior data allocation for the different types.

Data Type	Unclassified	Type 1	Type 2	Type 3	Type 4	Type 5	Type 6
Total data	1400	255	279	171	114	255	326
Total training data	980	179	195	120	80	179	228
Total test data	420	77	84	51	34	77	98

All sample data were standardized, and then Keras was used to complete DNN training and testing. Considering the limited data sample size of types 1–6, the number of iterations for each type in the model training process is 100. The scale of the unclassified data is large, and the number of iterations is set to 1000.

### 4.3 Test result of the decision model

**Fig 13:**
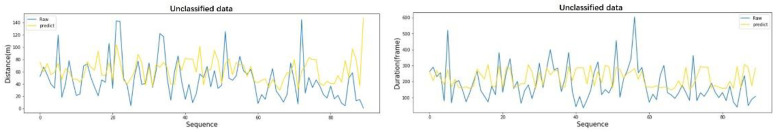
Prediction results of the weaving model without classification.

**Fig 14:**
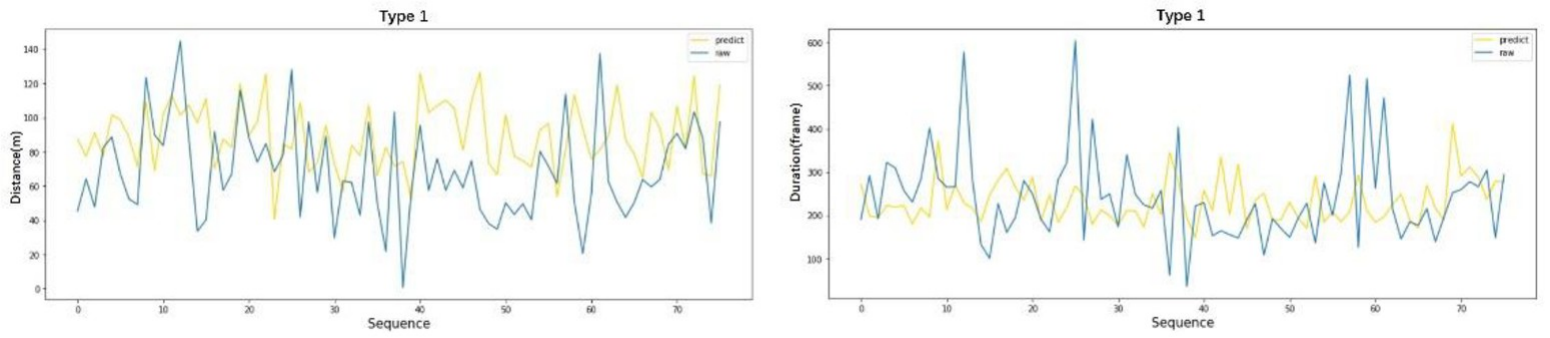
Prediction results of the weaving model for type 1.

**Fig 15:**
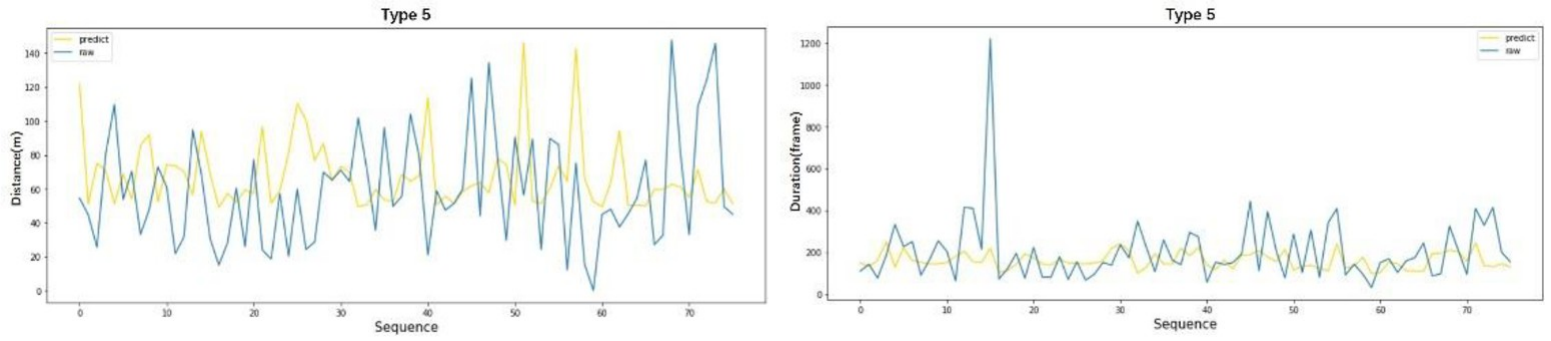
Prediction results of the weaving model for type 5.

To intuitively display the prediction results of the model, the prediction results of the unclassified data from type 1 and type 5 were extracted, and deviation analysis was conducted. The test dataset of the unclassified data is large, so only 90 of them are selected for deviation analysis, as shown in Fig 13. The deviation analyses of the type 1 and type 5 data is shown in Figs 14 and 15, respectively. The yellow line represents the predicted values for the lane-changing duration and distance, and the blue line represents the real values of the two parameters. As seen from the deviation distribution diagram, the DNN model has a high fitting degree for all types of weaving behaviors, which makes it difficult to directly evaluate the forecast results.

Through model training, it can be found that the MSE loss value decreases with the increase in the iterative number. When the iterative number exceeds the specified number, the decline rate of the loss value slows down significantly. The loss value of each type of weaving behavior under the specified iterative number is finally obtained, as shown in Table 3.

**Table 3:** The loss value of lane-changing duration and distance prediction results.

Distance Prediction	Data Type	Unclassified	Type 1	Type 2	Type 3	Type 4	Type 5	Type 6
	Loss	8.51	1.03	2.37	3.30	1.08	3.14	2.14
Duration Prediction	Data Type	Unclassified	Type 1	Type 2	Type 3	Type 4	Type 5	Type 6
	Loss	9.40	2.23	4.02	2.80	1.68	3.25	2.06

The following conclusions can be drawn from the above table:

- The prediction loss values of the lane-changing distance from type 1 to type 6 are 1.0 to 3.3, and the prediction loss values of the lane-changing duration are 1.6 to 4.0. The prediction results of the different types of lane-changing behaviors are very similar to the results after lane-changing behavior classification.
- By comparing the prediction results of the 6 types of lane-changing behaviors, it can be found that the prediction accuracies of type 1 and type 4 are the highest. The prediction accuracy of the lane-changing distance of type 1 is at least 51% higher than that of the other types. The prediction accuracy of lane-changing distance of type 4 is at least 49% higher than that of the other types, while the prediction accuracy of lane-changing duration is at least 18% higher than that of the other types. It can be concluded that the lane-changing behavior characteristics of vehicles are easier to predict when the lag distance is large or small, and the randomness of the lane-changing behavior characteristics of vehicles in the other cases is stronger.
- Although the data size of the unclassified lane-changing behavior is large and its iterative number is large, the prediction results of both the lane-changing duration and distance are not as good as the prediction results after classification. The prediction accuracy of the lane-changing distance is improved by at least 61% after classification, and the prediction accuracy of the lane-changing duration is improved by at least 57% after classification. This shows that the proposed classification method for weaving behavior can describe the characteristics of lane-changing behavior more accurately.

## 5. Discussion and conclusion

This paper studied the features and decision behavior of the lane-changing process in intertunnel weaving sections. Different from existing studies, lane-changing behavior was not treated as an instantaneous event, and the lane-changing process is the focus of this study. Weaving vehicles in the intertunnel weaving section were recorded, and trajectory data were collected, including the lane-changing duration, the lane-changing distance, and other information about the vehicle status. To analyze the difference in the lane-changing behavior characteristics in the different lane-changing environments, the lane-changing environments were classified, and decision modeling for different types of weaving behaviors before and after classification was carried out by a deep neural network. The following conclusions can be drawn based on the conducted analysis:

- The lane-changing duration has a lognormal distribution, and the lane-changing distance has a normal distribution.
- Taking the lead and lag distances at the beginning of lane-changing as the criteria, lane-changing environments can be divided into 6 types. From the perspective of the transverse distribution of the weaving points, the lane-changing behavior is mainly distributed in the outermost lane of the expressway and the inmost lane of the ground road. From the longitudinal distribution of the weaving points, the lane-changing points of types 1 to 4 are mainly in the first half of the weaving section, while the distributions of the points of types 5 and 6 are relatively uniform.
- There are significant differences among the different types of weaving behaviors in terms of the number of lane changes, distributions in the different lanes, distribution in the same lane, and statistical characteristics of the lane-changing behaviors. The lane-changing durations and distances of types 5 and 6 are discrete, while the two features of the other 4 types are concentrated.
- Through the decision modeling of different types of weaving behavior, it is found that the prediction accuracy of different types of lane-changing behavior is very similar to the accuracy after lane-changing behavior classification. Although the data size and the iterative number of the unclassified lane-changing behaviors are larger, the prediction results of both the lane-changing duration and distance are inferior to the prediction results after classification. The prediction accuracy of the lane-changing distance is improved by at least 61% after classification, and the prediction accuracy of the lane-changing duration is improved by at least 57% after classification.
- Among the prediction results of the six types of lane-changing behavior, the prediction accuracies of type 1 and type 4 are the highest. It can be seen that the lane-changing behavior characteristics of vehicles with large or small lag distances are easier to predict, while the randomness of the lane-changing behavior characteristics of vehicles in the other cases is stronger.

Considering that it usually takes more than one second to complete a merging movement, there is a critical need to model the merging behavior during the entire merging implementation period from the starting time of a merging maneuver to the completion time of the maneuver. The rules extracted from the merging behavior during the merging implementation period can be applied to the merging decision assistance system to improve system performance.

Along the stream of this study, several elements of future research can be identified. (1) The traffic flow was recorded by UAV and the traffic data was acquired through post-processing in the study. Data acquisition methods may reduce the accuracy of the data. More accurate traffic data would make our conclusions stronger. (2) This study only focuses on the difference of lane changing behavior. The causes of differential lane changing behavior and its impact on traffic flow have not been thoroughly discussed. And how to make use of differentiated lane changing behaviors under different environments for effective traffic control is still worth further research.

## Supporting information

S1 Data (RAR)
